# Impact of Age on Lipid-Lowering Therapy Prescriptions and LDL-Cholesterol Control: Insights from the PORTRAIT-DYS Study

**DOI:** 10.5334/gh.1543

**Published:** 2026-03-27

**Authors:** Cristina Gavina, Daniel Seabra, Sílvia Oliveira, Carla Teixeira, Jorge A. Ruivo, Nuno Lourenço-Silva, Ana Rita Luz, Cristina Jácome, Francisco Araújo

**Affiliations:** 1 Cardiology Department, ULS Matosinhos, 4464–513 Senhora da Hora, Matosinhos, Portugal; 2 Department of Medicine, Faculty of Medicine, University of Porto, Porto, Portugal; 3 UnIC, Faculty of Medicine, University of Porto, Porto, Portugal; 4 Department of Physiology and Cardiac Surgery, Faculty of Medicine, University of Porto, Porto, Portugal; 5 Medical Affairs, Daiichi-Sankyo, Porto Salvo, Portugal; 6 Department of Medicine, Lisbon Medical School, University of Lisbon, Lisbon, Portugal; 7 Centro Cardiovascular da Universidade de Lisboa, University of Lisbon, Lisbon, Portugal; 8 Department of Community Medicine, Information and Decision in Health, Faculty of Medicine, University of Porto, 4050–313 Porto, Portugal; 9 MTG Research and Development Lab, 4200–604 Porto, Portugal; 10 RISE-Health, MEDCIDS-Department of Community Medicine, Information and Health Decision Sciences, Faculty of Medicine, University of Porto, Porto, Portugal; 11 Department of Internal Medicine, Hospital Lusíadas, Lisbon, Portugal

**Keywords:** LDL-C management, Dyslipidemia, Real-world evidence, Primary care, Secondary care

## Abstract

**Background:**

Despite clear guidelines for lipid-lowering therapies (LLT) for patients with high and very-high atherosclerotic cardiovascular disease (ASCVD) risk, a significant gap persists between recommended low-density lipoprotein cholesterol (LDL-C) management and actual clinical practice, leaving a large proportion of patients without adequate treatment. Patient age is one of several factors that may play a role in this gap.

**Objectives:**

We characterized LLT prescription patterns among middle-aged and older adults with high and very-high ASCVD risk and estimated the effect of age in achieving LDL-C control.

**Methods:**

This cohort study used electronic health records of a Portuguese healthcare institution from January 2012 to December 2022. Middle-aged (40–69 years) and older (70–85 years) patients with high and very-high ASCVD risk were analysed. Exposure consisted of LLT prescriptions. LLT prescriptions were characterized in six dynamic patterns based on statin intensity (high, moderate, low) and the addition of ezetimibe. Risk for LDL-C control at 150 and 360 days was compared between the two age cohorts using multivariate Cox regressions.

**Results:**

A total of 36,866 patients were identified, accounting for 407,500 LLT prescriptions. High-intensity statins were more frequently prescribed to middle-aged patients (12.0% vs. 8.6% older), whereas older patients more often received low-intensity statins (8.6% vs. 5.9% middle-aged). The use of statin-ezetimibe combinations was low across all age groups (0.1–1.4%). At 150 and 360 days of follow-up, LDL-C targets were achieved in 2,661 (0.7%) and 14,047 (3.8%) prescriptions, respectively. Older patients had a 32% higher rate of reaching LDL-C goals at 150 days (HR = 1.32, 95% CI = 1.19–1.45) and 27% at 360 days (HR = 1.27, 95% CI = 1.19–1.35).

**Conclusion:**

LDL-C control remains low in high- and very-high-risk patients from a Portuguese integrated health care unit, with particularly low achievement rates among middle-aged adults, despite their higher use of high-intensity LLT. These findings highlight the need for optimized treatment strategies, including age-specific approaches.

## Introduction

Atherosclerotic cardiovascular disease (ASCVD) is one of the leading causes of morbidity and mortality worldwide (1). Low-density lipoprotein cholesterol (LDL-C) has been shown to be a causal factor and a central agent in the development of atherosclerosis (2,3,4). The linear relationship between elevated LDL-C and ASCVD has been well documented in numerous studies (1,2,5,6,7,8,9,10,11,12,13).

Effective LDL-C management through lipid-lowering therapies (LLT) is essential for reducing cardiovascular (CV) risk, particularly in high- and very-high-risk populations. The World Health Organization has established a target that at least 50% of eligible people receive drug therapy and counselling (including statins) to prevent major CV events such as myocardial infarction and stroke (14). Lowering LDL-C levels has proven beneficial in both primary and secondary prevention of ASCVD (3,8,15,16). According to the European Society of Cardiology (ESC) and European Atherosclerosis Society (EAS) guidelines, individuals at the highest risk of atherothrombotic events derive the greatest benefit from LDL-C reduction, with no minimum threshold for clinical efficacy (2,17,18).

Despite the compelling evidence and clear guidelines, real-world data consistently reveal a significant gap between recommendations and actual clinical practice regarding LLT prescription. Globally, a substantial proportion of patients eligible for LLT, especially those at high and very high risk, are not prescribed appropriate therapy. For instance, a systematic review indicated wide variability in prescription patterns across Europe, with high or very-high CV risk patients receiving high-intensity statins/LLT in a range from 1% to 97% and moderate-intensity therapy from 3% to 77% (19).

In addition, although the well-defined LDL-C targets for high- and very-high-risk patients are outlined in the 2019 ESC/EAS dyslipidemia management guidelines, these goals remain unmet in 70–80% of individuals, as reported in several European observational studies (20,21,22,23,24,25,26,27,28,29). Several factors contribute to this gap, including persistent inertia in prescribing and titrating statins (30,31) and a progressive decline in LLT adherence over time. This decline is influenced by patient-related factors such as age, socioeconomic status, drug intolerance, adverse effects, and comorbidities (32). This challenge is particularly pronounced in older adults, who often present with multiple comorbidities that complicate dyslipidemia management (2,33). Conversely, younger populations, while generally at lower short-term risk, may experience prolonged exposure to elevated LDL-C levels, significantly increasing their lifetime ASCVD risk (2,3,8).

Given these considerations, understanding LLT prescription patterns in high- and very-high-risk populations, as well as evaluating the effectiveness of these therapies across different age groups, is crucial.

This study aimed to characterize LLT prescribing trends among middle-aged and older adults with high and very-high ASCVD risk and to assess the impact of age on LDL-C control in a broad, real-world, unselected Portuguese population.

## Materials and Methods

### Study design

This is an observational retrospective cohort study using electronic health records (EHR) from the Unidade Local de Saúde de Matosinhos (ULSM), a healthcare institution comprising 14 primary care centres and one hospital that provides secondary and tertiary care services in the Matosinhos region in Portugal. The study used the EHR of middle-aged and older adults treated at ULSM between 1 January 2012 and 31 December 2022.

All methods were performed in accordance with the ethical guidelines for human participants. The Ethical Committee of the ULSM approved the study and waived the requirement for informed consent (18/CES/JAS). This study was reported following the recommendations for reproducibility and validity assessment for healthcare database studies (34).

### Study population

Two age cohorts were defined (middle-aged adults, 40–69 years; older adults, 70–85 years), and the index date was defined as the time when an LLT was prescribed. Patients were included if the following criteria were met simultaneously: (i) age between 40 and 85 years; (ii) at least one appointment with a ULSM primary care physician in the three years preceding the index date, in line with the Portuguese official government indicator used to determine whether a patient is routinely followed or not; (iii) at least one record in the last year before the index date; (iv) have an LLT prescription; and (v) have a high or very-high CV risk defined according to the ESC/EAS guidelines 2019 (Supplementary Table S1) (2). The inclusion criteria were designed to ensure maximum overlap between the study population and the resident population within the selected age group. This approach was partly based on the 2021 Portuguese Census, which indicates that the study population accounted for approximately 90% of the local resident population of Matosinhos—the eighth most inhabited municipality in Portugal and the fourth in the northern region (35).

### Exposure

Exposure was defined as any time point at which an LLT drug was prescribed (regardless of duration). Prescription data, registered according to the Anatomical Therapeutic Chemical (ATC) Classification System (36) and corresponding dosage information, were used to compute LLT categories (Supplementary Tables S2 and S3). The statin intensity group, considering both the drug and the dosage, was classified according to the American College of Cardiology and the American Heart Association (37). LLT prescriptions were characterized in six dynamic patterns defined based on statin intensity (high, moderate, and low) and the addition of ezetimibe to each intensity group: low-intensity statin, moderate-intensity statin, high-intensity statin, low-intensity statin + ezetimibe, moderate-intensity statin + ezetimibe, and high-intensity statin + ezetimibe. Prescriptions of fixed or single-pill combinations of the LLTs were considered.

We used an episode-based approach, allowing patients who met all eligibility criteria to become eligible for multiple LLT prescription patterns during the index period, as they could have multiple LLT prescriptions over time. When a patient who was already being followed under one LLT prescription pattern became eligible for another LLT prescription pattern, they were followed in the new prescription pattern while continuing follow-up in the former. In case a patient switched statins or doses within the same intensity range, it would remain in the same LLT prescription pattern.

### Outcome

Since distinct LDL-C targets were defined between 2012 and 2022, we considered LDL-C target achievement as meeting the risk-based goals outlined in the contemporaneous ESC/EAS guidelines (2012–2016 (38), 2016–2019 (16), 2020–2022 (2); Supplementary Table S4). A minimum time frame of 150 days was chosen to ensure treatment effects (39). In addition, this time frame is within the follow-up window of 60–180 days recommended by the Portuguese National Health Service to reevaluate patients after LLT is initiated or its intensity is changed. However, recognizing that clinical practice can vary, we also assessed LDL-C target achievement up to 360 days (150, 180, 210, 240, 270, 300, 330, 360 days).

### Other variables

The patient’s age and sex were determined from the administrative record available in the EHR. The presence of other health conditions, such as alcohol or drug abuse, atrial fibrillation, estimated glomerular filtration rate <60 mL/min, heart failure, hypertension, mental health disorder, myocardial infarction, obesity, peripheral artery disease, primary hypertriglyceridemia, stroke, type 1 and type 2 diabetes mellitus, and unstable angina, was defined using the most comprehensive and granular records available, which included (i) visit and diagnosis data, which were coded using the International Classification of Primary Care, 2nd edition (ICPC-2), the International Classification of Disease, 9th revision (ICD-9), and the International Classification of Disease, 10th revision (ICD-10) codes; (ii) laboratory and clinical measurements, coded using ad hoc vocabularies that were standardized to the Systematized Nomenclature of Medicine Clinical Terms (SNOMED CT); (iii) prescribed medications, coded using the ATC Classification System; and (iv) free-text clinical notes from both primary and secondary care. A detailed definition of these variables is shown in Supplementary Table S5.

### Approach to data processing

Data processing was performed using an algorithm-to-data approach that removes the need to share patient-level data with researchers. This means that all relevant data processing steps were developed prior to data processing and made available in a study package for ULSM to execute locally. The study package for this study was developed using SIGIL Scientific Enterprises Vero Framework and was compiled for the ULSM infrastructure based on Apache Trino 466. The study package implemented the complete data engineering pipeline to process data in the Observational Medical Outcomes Partnership (OMOP) Common Data Model (CDM) version 5.4 (40) format into aggregate statistical reports in tabular format.

ULSM maintains a data lakehouse that contains a complete copy of all EHR records, structurally formatted according to OMOP CDM. For this study, semantic mappings into standard concepts of OMOP CDM were not used. Only original vocabularies from source systems were used to derive working definitions for variables of interest.

### Statistical analysis

The analysis considered an episode-based design, where exposure consists of any time when LLT was prescribed. Descriptive statistics were used to present the global characteristics (age, sex, CV risk, LDL-C, and LDL-C control) of the study population at the time of entering the cohort (baseline), categorized into six LLT prescription patterns. The continuous variables were reported as the median (P50), 25th percentile (P25), and 75th percentile (P75). Categorical variables were presented as absolute and relative frequencies. The incidence of LDL-C control events was measured as events per 100 patient-years (100 PY) to evaluate treatment efficacy. Risk for LDL-C control was compared between middle-aged (40–69 years) and older (70–85 years), using a Cox proportional hazards model for each follow-up time (150, 180, 210, 240, 270, 300, 330, 360 days) adjusted at baseline for sex, LLT prescription patterns (low-intensity statin, moderate-intensity statin, high-intensity statin, low-intensity statin + ezetimibe, moderate-intensity statin + ezetimibe, and high-intensity statin + ezetimibe), and comorbidities (alcohol or drug abuse, atrial fibrillation, estimated glomerular filtration rate <60 mL/min, heart failure, hypertension, mental health disorder, myocardial infarction, obesity, peripheral artery disease, primary hypertriglyceridemia, stroke, type 1 and type 2 diabetes mellitus, and unstable angina). No missing data of any variables were imputed for the analysis. We used Apache Trino v. 464 to engineer the final dataset, R v. 4.3.2 to perform the statistical analysis using the Survival package (41), and Vega-lite to generate figures (42).

## Results

### Study population

A total of 36,866 patients with 407,500 episodes of LLT prescription were identified between 1 January 2012 and 31 December 2022. Baseline characteristics of the patients at the time of the first LLT prescription are shown in Table 1. On average, each patient contributed to 11.1 eligible episodes of LLT prescription. Most episodes were from middle-aged (n = 230,171; 56.5%), male (n = 216,347; 53.1%), and very-high-risk (n = 375,547; 92.2%) patients.

**Table 1:** Patient characteristics at the time of the first lipid-lowering therapy (LLT) prescription.


	LOW INTENSITY		MODERATE INTENSITY		HIGH INTENSITY		LOW INTENSITY + EZETIMIBE		MODERATE INTENSITY + EZETIMIBE		HIGH INTENSITY + EZETIMIBE	

Episodes (n)	2,343		17,800		7,573		112		1,525		1,205	

Age (years, P50, P25–P75)	66.3	57.3–73.7	63.4	54.9–71.6	64.9	57.3–72.1	68.8	59.8–74.5	66.4	58.7–73.1	65.3	58.2–72.0

**Age cohorts (years), n (%)**												

40–69	1,478	(63.1%)	12,560	(70.6%)	5,157	(68.1%)	63	(56.2%)	972	(63.7%)	823	(68.3%)

70–85	865	(36.9%)	5,240	(29.4%)	2,416	(31.9%)	49	(43.8%)	553	(36.3%)	382	(31.7%)

Female, n (%)	1,166	(49.8%)	7,897	(44.4%)	3,126	(41.3%)	55	(49.1%)	709	(46.5%)	451	(37.4%)

**CV risk level, n (%)**												

Very-high risk	2,095	(89.4%)	15,816	(88.9%)	7,229	(95.5%)	107	(95.5%)	1,432	(93.9%)	1,182	(98.1%)

High risk	248	(10.6%)	1,984	(11.2%)	344	(4.5%)	<5	(Masked)	93	(6.1%)	23	(1.9%)

LDL-C control^a^, n (%)	135	(5.8%)	794	(4.5%)	299	(4.0%)	<5	(Masked)	106	(7.0%)	56	(4.7%)

**Comorbidities**												

Obesity	630	(26.9%)	4,842	(27.2%)	2,220	(29.3%)	31	(27.7%)	491	(32.2%)	380	(31.5%)

Hypertension	1,846	(78.8%)	13,249	(74.4%)	5,876	(77.6%)	92	(82.1%)	1,246	(81.7%)	976	(81.0%)

Alcohol or drug abuse	293	(12.5%)	2,555	(14.4%)	1,547	(20.4%)	14	(12.5%)	258	(16.9%)	292	(24.2%)

Type 1 diabetes mellitus	120	(5.1%)	847	(4.8%)	529	(7.0%)	≤5	(Masked)	121	(7.9%)	97	(8.0%)

Type 2 diabetes mellitus	1,419	(60.6%)	10,702	(60.1%)	4,812	(63.5%)	59	(52.7%)	983	(64.5%)	786	(65.2%)

Atrial fibrillation	162	(6.9%)	1,125	(6.3%)	613	(8.1%)	9	(8.0%)	112	(7.3%)	110	(9.1%)

Primary hypertriglyceridemia	227	(9.7%)	2,006	(11.3%)	1,200	(15.8%)	22	(19.6%)	300	(19.7%)	239	(19.8%)

Stroke	226	(9.6%)	1,873	(10.5%)	1,247	(16.5%)	8	(7.1%)	167	(11.0%)	194	(16.1%)

Myocardial infarction	123	(5.2%)	1,447	(8.1%)	1,624	(21.4%)	11	(9.8%)	200	(13.1%)	416	(34.5%)

Peripheral artery disease	156	(6.7%)	1,053	(5.9%)	893	(11.8%)	10	(8.9%)	148	(9.7%)	187	(15.5%)

Unstable angina	73	(3.1%)	706	(4.0%)	791	(10.4%)	9	(8.0%)	121	(7.9%)	236	(19.6%)

Heart failure	162	(6.9%)	1,206	(6.8%)	890	(11.8%)	8	(7.1%)	154	(10.1%)	206	(17.1%)

Mental health disorder	841	(35.9%)	6,084	(34.2%)	3,520	(46.5%)	58	(51.8%)	695	(45.6%)	602	(50.0%)

Estimated glomerular filtration rate <60 mL/min	324	(13.8%)	1,875	(10.5%)	1,153	(15.2%)	19	(17.0%)	299	(19.6%)	223	(18.5%)


Abbreviations: CV: cardiovascular; LDL-C: low-density lipoprotein cholesterol; P50: median; P25: 25th percentile; P75: 75th percentile. ^a^Adjusted control by ESC 2011, 2016, and 2019 guidelines.

### LLT prescription patterns

The distribution of episodes of LLT prescription across the two age cohorts is presented in Table 2. Most episodes identified were related to the prescription of statins in monotherapy: moderate intensity (80.0%), high intensity (10.3%), and low intensity (7.0%). In both age cohorts, statins in monotherapy (80.0% moderate, 10.5% high, and 7.0% low intensity) were more commonly prescribed than statin-ezetimibe combinations (1.4% moderate, 1.0% high, and 0.1% low intensity).

**Table 2:** Baseline lipid-lowering therapy prescription patterns for the total cohort and the two age cohorts.


	TOTAL COHORT(n = 407,500)		AGE COHORTS, n(%)			

			40–69 YEARS (n = 230,171)		70–85 YEARS (n = 177,329)	

Low-intensity statin, n (%)	28,728	(7.0%)	13,504	(5.9%)	15,224	(8.6%)

Moderate-intensity statin, n (%)	325,804	(80.0%)	183,028	(79.5%)	142,776	(80.5%)

High-intensity statin, n (%)	42,785	(10.5%)	27,581	(12.0%)	15,204	(8.6%)

Low-intensity statin + ezetimibe, n (%)	420	(0.1%)	193	(0.1%)	227	(0.1%)

Moderate-intensity statin + ezetimibe, n (%)	5,728	(1.4%)	3,221	(1.4%)	2,507	(1.4%)

High-intensity statin + ezetimibe, n (%)	4,035	(1.0%)	2,644	(1.1%)	1,391	(0.8%)


The use of moderate-intensity statins in monotherapy was similar across age cohorts (middle-aged 79.5%, older 80.5%), but patients in the middle-aged cohort were more frequently prescribed high intensity (12.0% vs. older 8.6%), while older patients were more frequently prescribed low intensity (8.6% vs. middle-aged 5.9%). The prescription of statin-ezetimibe combinations was similar across age cohorts.

### Risk for LDL-C control

**Figure 1:**
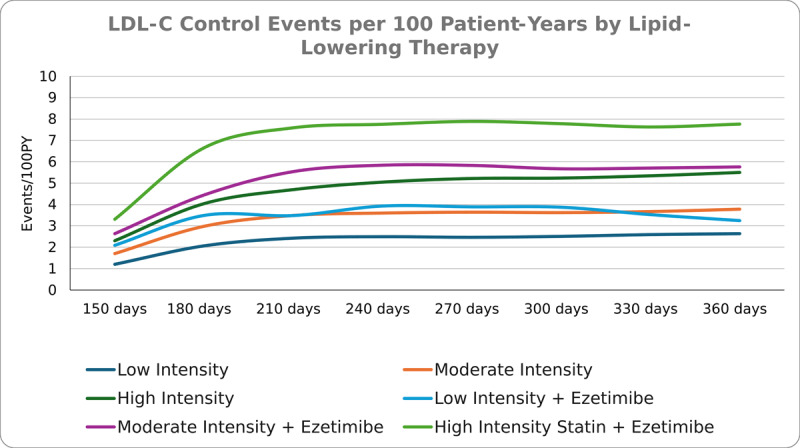
Rate of low-density lipoprotein-cholesterol (LDL-C) control events per 100 patient-years by lipid-lowering therapy (LLT) during follow-up.

Considering 369,755 episodes, at 150 days of follow-up, LDL-C control was reached for 2,661 (0.7%) episodes of LLT prescription. LDL-C control increased during follow-up, up to 14,047 (3.8%) episodes at 360 days. This increasing trend was seen in all six LLT prescription patterns (Figure 1, Supplementary Table S6).

**Figure 2:**
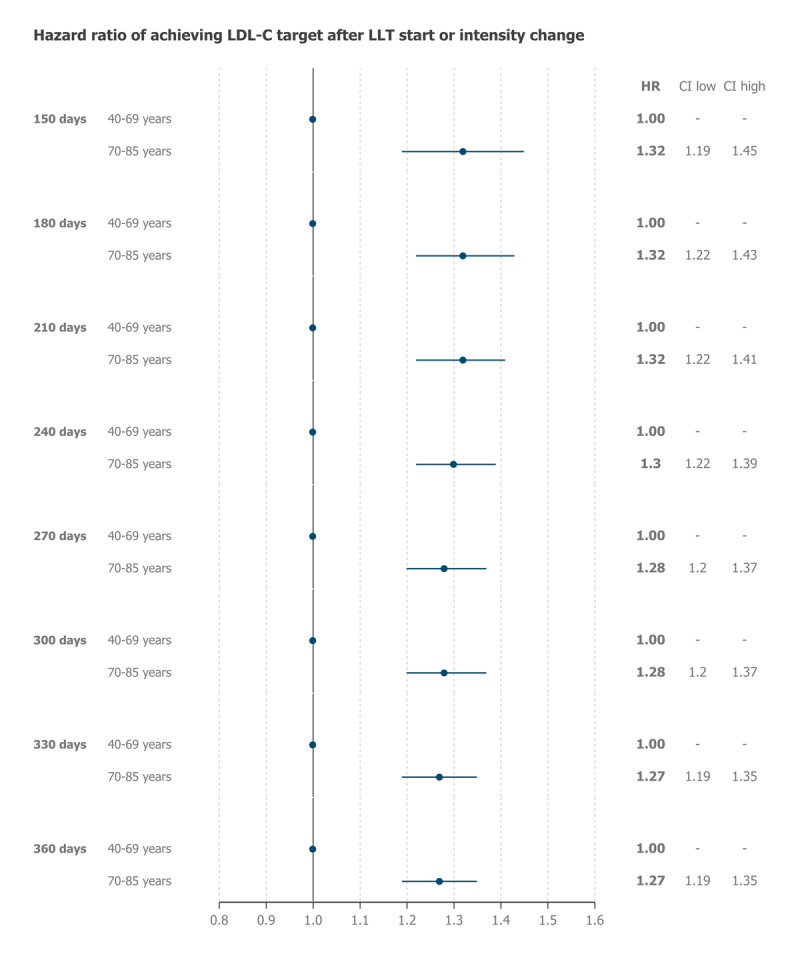
Adjusted hazard ratios of achieving low-density lipoprotein cholesterol (LDL-C) control target following lipid-lowering therapy (LLT) prescription episodes in older patients (70–85 years), using episodes from middle-aged patients (40–69 years) as the reference.

According to the regression model, episodes of LLT prescription in older patients were associated with a 32% higher rate of achieving the LDL-C goal at 150 days of follow-up (HR = 1.32, 95% CI = 1.19–1.45) compared to middle-aged patients (Figure 2). This rate varied slightly (from 27% to 32%) and remained significant at all follow-up times (Figure 2). Regression models for each follow-up time are provided in Supplementary Tables S7–S14.

## Discussion

This study highlights significant age-related differences in hypercholesterolemia management. Episodes of LLT prescription from older adults had higher rates of achieving LDL-C targets following LLT prescription compared to those from middle-aged patients, regardless of LLT intensity, CV risk category, sex, or comorbidities.

Supporting our findings, Karlson et al. analysed individual patient data from the VOYAGER database to assess the impact of statin therapy on LDL-C levels in patients aged ≥70 years. Their analysis demonstrated a 2.7% greater LDL-C reduction in older patients compared to younger ones (43,44). This effect remained consistent across different statins and doses, with no significant interactions between age and gender. Notably, the age-related effects were similar for both men and women, and sex-related effects remained consistent across all age groups (43). While the underlying mechanisms for these age-based discrepancies remain to be fully elucidated, we hypothesized that they may stem from pharmacokinetic shifts (43) or survival bias, such as reduced dietary cholesterol intake, higher medication adherence, and more frequent clinical follow-up in older populations. Nevertheless, further investigation is required. Yet, we must emphasize that LDL-C control remained consistently low across all age groups in this study (2012–2022), with only 0.7% of episodes reaching target levels at 150 days and 3.8% at 360 days. This finding aligns with previous episode-based research in this specific population, which reported LDL-C control rates of 3.4–7.1% among high- and very-high-risk patients during 2020 (45). Broader European studies report higher goal achievement rates ranging from 17% to 22% (46,47). However, direct comparisons between these results are confounded by the fundamental differences between episode-based and patient-based methodologies. In addition, analysing a ten-year period may mask any gradual improvements in LDL-C control that have occurred, particularly since the release of the 2019 ESC guidelines, which introduced more stringent targets to lipid lowering (2). Future studies could therefore focus on LDL-C control post-2019 ESC guidelines to accurately assess if this trend is improving.

Multiple studies have highlighted the importance of LDL-C reduction in older adults. A 2019 meta-analysis found that in individuals over 75 years, each 1 mmol/L reduction in LDL-C was associated with a significantly lower risk of major vascular events (RR = 0.82, 95% CI = 0.70–0.95) and major coronary events (RR = 0.82, 95% CI = 0.70–0.96) (48). Statin therapy effectively reduces major vascular events across all age groups, with strong evidence supporting its use in older adults at high risk of CV events. Maycock et al. found that in patients with coronary artery disease, statin use led to a 50% reduction in mortality among those aged 80 years and older, compared to a 44% reduction in individuals aged 65–79 years and a 30% reduction in those under 65 years, even after adjusting for potential confounders (49). Similarly, a 2021 meta-analysis by Awad et al. demonstrated that in adults aged ≥65 years without prior cardiovascular disease (CVD), statin therapy was associated with a significantly lower risk of all-cause mortality (14% reduction), CV mortality (20% reduction), and stroke (15% reduction) (50). Subgroup analysis confirmed a significant association between statin use and reduced all-cause mortality across all age groups: 65–75 years (HR = 0.84, 95% CI = 0.81–0.88), ≥75 years (HR = 0.88, 95% CI = 0.81–0.96), ≥80 years (HR = 0.84, 95% CI = 0.79–0.89), and ≥85 years (HR = 0.88, 95% CI = 0.79–0.99) (50). These findings are consistent with CV outcome studies showing that the absolute reduction in CV event rates with statin therapy is greater in older patients than in younger individuals. Moreover, the benefits of treatment tend to emerge soon after statin initiation (51,52). Our study found that older adults were primarily represented in the low-intensity statin and low-intensity statin plus ezetimibe groups, whereas middle-aged patients were predominant in other LLT categories. This aligns with previous research showing that, despite having higher LDL-C levels, older adults are less frequently prescribed statins, particularly high-intensity therapies, compared to younger individuals. Nanna et al. demonstrated that older adults (>75 years) with prior ASCVD were less likely to receive any statin (80.1% vs. 84.2%; p = 0.003) and significantly less likely to be prescribed high-intensity statins (23.5% vs. 36.2%; p < 0.0001) compared to younger adults (53). Additionally, studies from the United Kingdom, the United States, and the Netherlands conducted between the late 1990s and early 2010s indicate that LLT prescriptions decline after age 75, regardless of prior CVD history (54,55,56). But, even with lower-intensity LLT, older adults in our cohort demonstrated superior LDL-C control than middle-aged adults. The very low uptake of ezetimibe observed in our cohort probably reflects a ‘statin-only’ prescribing culture. This underutilization of ezetimibe is documented in other real-world studies (57,58,59), though our findings are more pronounced. This underscores a persistent therapeutic gap where ezetimibe could potentially bridge the distance to LDL-C goals in older patients who are often restricted to low-intensity statin regimens.

The differences in prescription rates across age groups highlight the need for closer examination. Therapeutic inertia in older adults may stem from their underrepresentation in randomized clinical trials and concerns about increased risks of adverse events and drug-drug interactions with ageing (60). Additionally, factors such as limited life expectancy, multiple comorbidities, polypharmacy, and altered drug metabolism present significant challenges in achieving optimal LDL-C control in this population (61).

Addressing concerns about statin use in older patients is essential. Jung et al. confirmed that the increased ASCVD risk associated with high LDL-C persists in individuals aged ≥75 years, reinforcing the importance of LDL-C control across all age groups (61). The 2021 ESC Guidelines on Cardiovascular Disease Prevention (1) strongly recommend statin therapy for older adults (≥70 years) with ASCVD (Class I, Level A). For primary prevention, statin initiation may be considered in high- or very-high-risk individuals over 70 (Class IIb, Level B), with lower starting doses advised in cases of renal impairment or potential drug interactions (Class I, Level C). A tailored approach is crucial for optimizing CV disease prevention in older adults. Further research should explore age-related factors affecting LLT goal attainment, including pharmacokinetics, pharmacodynamics, comorbidities, and adherence challenges. Understanding these aspects will help refine treatment strategies and improve CV outcomes in this population.

Our study also demonstrates that males were more likely to achieve LDL-C targets compared to females, a finding that aligns with previous research utilizing ULSM data (39) as well as international evidence (62,63,64). Several clinical explanations have been proposed for this sex-based discrepancy, including lower medication adherence and higher rates of therapy discontinuation among women (39). While the current study could not definitively account for these behavioural factors, this finding underscores the critical need for further investigation and the development of sex-tailored management strategies in LLT.

This study yields important information on the trends of prescription of LLT on a large cohort with limited selection bias, loss to follow-up, and a small amount of missing data. Considering the high usage rate of ULSM by the resident population, the low population migration rates, and the large data collection period, the authors believe that these findings can be generalized to the population served in this region and to populations of comparable profile. The statistical analysis approach based on LLT episodes, allowing patients to be followed in more than one cohort, not censoring on new cohort inclusion, and including covariates that frequently account for the competing risk of death, enabled a conservative estimation of risks, which were still shown to be of high clinical magnitude. This study incorporated contemporary CV risk definitions and LDL-C targets from current guidelines, thereby reducing potential biases associated with changes in guidelines over time. By concentrating on patients prescribed LLT and employing an episode-based approach to model multiple prescriptions over time, the study effectively estimates the rate of achieving LDL-C goals.

Still, there are limitations in this study that need to be acknowledged. ULSM serves a predominantly urban population with broad primary healthcare coverage and thus may not be representative of other regions of Portugal. This analysis was based on retrospective EHR data with their unavoidable potential for quality and completeness issues and thus vulnerable to bias or residual confounding that hinders causal inference. Specifically, the observed selection bias within the six LLT patterns, particularly the less frequent use of ezetimibe, is a recognized challenge in observational studies. Although we did not apply an inverse probability of treatment weighting approach in this study to explicitly address this, we acknowledge it as a robust methodology that could have further strengthened our findings against confounding by indication. This study could also have been strengthened by analysing patients’ adherence to LLT, as shifts in medication-taking behaviour might partially explain the observed differences between age cohorts. However, prescription refill data were not available in the ULSM data lakehouse. Furthermore, the study only considers patients between 40 and 85 years of age to whom LLT was prescribed, and therefore does not account for differences in control among patients who did not use LLTs. These limitations may limit the generalizability of the findings to the broader population of patients with dyslipidemia and should be addressed in future studies. Our study utilized an episode-based analysis, and multiple LLT episodes from the same patient were included in our Cox models, without adjustment for statistical dependence. This may have affected the precision of our estimates and should be considered a limitation of the current findings. Furthermore, the aggregation of data across multiple guidelines (2011, 2016, and 2019) may have introduced a temporal bias into our results. Future longitudinal studies should consider implementing sensitivity analyses—specifically stratifying by guideline era—to account for the progressive shift toward more stringent LDL-C targets.

## Conclusions

LDL-C control remains low in high- and very-high-risk patients from a Portuguese integrated health care unit, with particularly low achievement rates among middle-aged adults, despite their higher use of high-intensity LLT. In contrast, older adults had higher rates of achieving the LDL-C target, regardless of LLT intensity, CV risk category, sex, or comorbidities. Notably, this population had higher prescription rates of lower-intensity statins, both as monotherapy and in combination with ezetimibe. These findings suggest that despite receiving less intensive therapies, older adults still derive significant benefit from LLT in managing LDL-C levels. Future research should explore the underlying factors contributing to these prescribing patterns and develop age-specific strategies to optimize dyslipidemia management and reduce lifelong LDL-C exposure.

## Data Accessibility Statement

All aggregate statistical results are incorporated into the article and its online supplementary material. Patient-level data used in this study are not publicly available.

## Additional File

The additional file for this article can be found as follows:

10.5334/gh.1543.s1 Supplementary Material. Supplementary information including setting details, and tables with definitions of cardiovascular risk, lipid-lowering therapy categories, LDL-C targets per ESC/EAS guideline periods, study variable definitions, and regression model summaries for follow-up periods from 150 to 360 days (Tables S1–S14).
