# Supplementary material for: Impact of Age on Lipid-Lowering Therapy Prescriptions and LDL-Cholesterol Control: Insights from the PORTRAIT-DYS Study
Source: Glob Heart. 2026 Mar 27;21(1):29. doi: 10.5334/gh.1543 (PMC13025197; doi:10.5334/gh.1543)
Supplement: Supplementary Material. — Supplementary information including setting details, and tables with definitions of cardiovascular risk, lipid-lowering therapy categories, LDL-C targets per ESC/EAS guideline periods, study variable definitions, and regression model summaries for follow-up periods from 150 to 360 days (Tables S1–S14). [file gh-21-1-1543-s1.pdf]

Impact of age on lipid-lowering therapy prescriptions and LDL-cholesterol control: insights from the PORTRAIT-DYS study

Supplementary Material

Contents

Local Health Unit of Matosinhos constitution....2
ULSM Units....2
Supplementary tables....4
Table S1....4
Table S2....5
Table S3....6
Table S4....7
Table S5....8
Table S6....11
Table S7....14
Table S8....16
Table S9....18
Table S10....20
Table S11....22
Table S12....24
Table S13....26
Table S14....28

Local Health Unit of Matosinhos constitution

Local Health Unit of Matosinhos (ULSM) provides primary, differentiated and continuous healthcare. Its area of influence corresponds to the municipalities of Matosinhos, Vila do Conde and Póvoa de Varzim.

ULSM Units

- **Pedro Hispano Hospital**
- **Health Care Center Leça da Palmeira**
  - Family Health Unit Leça
  - Family Health Unit Maresia
  - Family Health Unit Dunas
  - Family Health Unit Progresso
  - Primary Care Unit Santa Cruz
  - Continuity Care Unit Leça
- **Health Care Center Matosinhos**
  - Family Health Unit Horizonte
  - Family Health Unit Oceanos
  - Primary Care Unit Matosinhos
  - Continuity Care Unit Matosinhos
- **Health Care Center Senhora da Hora**
  - Family Health Unit Caravela
  - Family Health Unit Lagoa
  - Family Health Unit Custóias
  - Continuity Care Unit Senhora da Hora

- **Health Care Center S. Mamede de Infesta**
    - Family Health Unit Infesta
    - Family Health Unit Porta do Sol
    - Primary Care Unit S. Mamede
    - Continuity Care Unit S. Mamede

Supplementary tables

Table S1

Definitions of risk according to 2019 ESC/EAS Guidelines

Variable	Definition
High risk	At least one is true:<br>1. TC > 310 mg/dL<br>2. LDL-C > 190 mg/dL<br>3. SBP ≥ 180mmHg or DBP ≥ 110mmHg twice ≥ 7 days<br>4. FH<br>5. DM with no target organ damage and either<br>a. Another CV Risk Factor<br>b. Duration ≥ 10 years<br>6. eGFR within [30, 60[mL/min<br>7. SCORE within [5, 10[%
Very-high risk	At least one is true:<br>1. MI, UA, SA, PCI, CABG, STK, TIA, or PAD<br>2. DM with target organ damage<br>3. At least 3 Major CV Risk Factors<br>4. eGFR < 30 mL/min<br>5. T1DM duration > 20 years<br>6. SCORE ESC 19 ≥ 10%<br>7. FH with either ASCVD or Major CV Risk Factor

ASCVD - atherosclerotic cardiovascular disease; CABG - coronary artery bypass graft surgery; CV - cardiovascular; DBP - diastolic blood pressure; DM - diabetes mellitus; eGFR: estimated glomerular filtration rate; EAS: European Atherosclerosis Society; ESC: European Society of Cardiology; FH: familial hypercholesterolemia; LDL-C: low-density lipoprotein cholesterol; MI: myocardial infarction; SBP: systolic blood pressure; SCORE: systematic coronary risk estimation; PAD: peripheral arterial disease; PCI: percutaneous coronary intervention; SA: stable angina; STK: stroke; T1DM, type 1 diabetes mellitus; T2DM, type 2 diabetes mellitus; TC: total cholesterol; TIA: transient ischemic attack; UA: unstable angina.

Table S2

Definitions of statin intensity categories (Grundy et al. 2019)

Variable	Definition
Low intensity statin	Simvastatin 10 mg or Pravastatin 10–20 mg or Lovastatin 20 mg or Fluvastatin 20–40 mg
Moderate intensity statin	Atorvastatin 10–20 mg or Rosuvastatin 5–10 mg or Simvastatin 20–40 mg or Pravastatin 40–80 mg or Lovastatin 40 mg or Fluvastatin XL 80 mg or Pitavastatin 1–4 mg
High intensity statin	Atorvastatin 40–80 mg or Rosuvastatin 20–40 mg

Table S3

Definitions of lipid lowering treatment categories

Variable	Definition
Statin	Patients prescribed with any statin
Low intensity statin	Patients prescribed with any low intensity statin
Moderate intensity statin	Patients prescribed with any moderate intensity statin
High intensity statin	Patients prescribed with any high intensity statin
Ezetimibe + Statin	Patients prescribed with ezetimibe plus any statin (low/moderate/high intensity statin)
Ezetimibe + Low intensity statin	Patients prescribed with ezetimibe plus low intensity statin
Ezetimibe + Moderate intensity statin	Patients prescribed with ezetimibe plus moderate intensity statin
Ezetimibe + High intensity statin	Patients prescribed with ezetimibe plus high intensity statin
Ezetimibe monotherapy	Patients prescribed with ezetimibe
Fibrates	Patients prescribed with fibrates

Table S4

Risk-based LDL-C goals according to ESC/EAS Guidelines 2019, 2016 and 2011.

Study period	ESC/EAS Guideline	Risk category	LDL-C goal
2020-2022	2019 ESC/EAS Guidelines	High	A goal of <70 mg/dL (<1.8 mmol/L) * and a >50% reduction from baseline
		Very-high	A goal of <55 mg/dL (<1.4 mmol/L) * and a >50% reduction from baseline
2016-2019	2016 ESC/EAS Guidelines	High	A goal of <100 mg/dL (<2.6 mmol/L) * or a >50% reduction from baseline if the baseline LDL-C is between 100 and 200 mg/dL (2.6 and 5.2 mmol/L)
		Very-high	A goal of <70 mg/dL (<1.8 mmol/L) * or a >50% reduction from baseline if the baseline LDL-C is between 70 and 135 mg/dL (1.8 and 3.5 mmol/L)
2012-2016	2011 ESC/EAS Guidelines	High	A goal of <100 mg/dL (<2.6 mmol/L)
		Very-high	A goal of <70 mg/dL (<1.8 mmol/L) * or a >50% LDL-C reduction when target level cannot be reached

EAS - European Atherosclerosis Society; ESC - European Society of Cardiology; LDL-C - low-density lipoprotein cholesterol; * Only absolute target levels were implemented.

Table S5

Study variables definitions

Variable	Definition
Age	Patient age in years
Alcohol or Drug Abuse	At least one is true:<br>• ICD-9: 305<br>• ICD-10: F101, F19<br>• ICPC-2: P15, P16, P19
Atherosclerotic disease	Meets criteria for UA, MI, ischemic STK, or PAD
Atrial fibrillation	At least one in true:<br>• ICD-9: 4273<br>• ICD-10: I48<br>• ICPC-2: K78
Cardiovascular disease	Meets criteria for SA, UA, MI, AF, STK, HTN, CKD, PAD, or TIA
Chronic kidney disease	At least one measurement of eGFR ≥ 60 mL/min followed by two measurements of eGFR < 60 mL/min, the latter at least 90 days apart. CKD is staged from the latest measurement
Coronary heart disease	Meets criteria for UA, MI, PCI, or CABG
CVD Risk level ESC 19	CVD Risk level as defined in 2019 European Cardiology Society Guidelines for Dyslipidaemias (Low Risk, Intermediate Risk, High Risk, Very High Risk)
Definite Familial hypercholesterolemia	At least one is true:<br>• ICD-9: 2720<br>• ICD-10: E7801
Familial hypercholesterolemia	Meets criteria for Definite FH or Possible FH
Glucose Lowering Drugs	ATC: A10
Haemorrhagic stroke	At least one is true:<br>• ICD-9: 43[0-2]<br>• ICD-10: I6[012]
Heart failure	At least one is true:<br>• ICD-9: 404[019]3, 428<br>• ICD-10: I110, I130, I132, I50

Variable	Definition
	● ICPC-2: K77
Hypertension	At least one is true:<br>● ICPC-2: K85, K86, K87<br>● SBP >140 mmHg or DBP >90 mmHg twice >7 days
Ischemic stroke	At least one is true:<br>● ICD-9: 43[3-7]<br>● ICD-10: I6[36]
LDL-C Control ESC 19	Patients with LDL-C levels under the recommended 2019 ESC/EAS target according with CVD risk level
Mental Health Disorder	At least one is true:<br>● ICD-9: 29[0-9], 30[0-2], 30[6-9], 31[0-9]<br>● ICD-10: F[0-9]<br>● ICPC-2: P70, P71, P72, P73, P74, P75, P76, P77, P78, P79, P80, P81, P82, P85, P86, P98, P99
Myocardial infarction	At least one is true:<br>● ICD-9: 410<br>● ICD-10: I21, I22, I256<br>● ICPC-2: K75
Obesity	Body mass index ≥ 30 kg/m2
Peripheral artery disease	At least one is true:<br>● ICD-9: 440, 441, 444<br>● ICD-10: I702, I739, I74[234589]<br>● ICPC-2: K92
Possible Familial hypercholesterolemia	At least one is true:<br>● ICD-10: Z8342<br>● All are true<br>○ TC > 290 mg/dL or LDL-C > 190 mg/dL<br>○ MI ≤ 60 years in first-degree relative<br>○ MI ≤ 50 years in second-degree relative<br>○ TC > 290 mg/dL in adult first- or second-degree relative
Primary hypertriglyceridemia	Triglycerides ≥ 175 mg/dL on two consecutive tests with a minimum of 120 days apart
SCORE	SCORE, ESC 19 (0%; 1%; [2, 5[%; [5, 10[%; [10,[%)
Sex	Patient sex (male/female)

Variable	Definition
Smoking	More recent smoking status (current smoker/former smoker/never smoked)
Stable angina	At least one is true:<br>● ICD-9: 413, 4140<br>● ICD-10: I201, I208, I209, I251, I255<br>● ICPC-2: K76
Stroke	At least one is true:<br>● ICPC-2: K89, K90<br>● Hemorrhagic or Ischemic Stroke
Transient ischemic attack	At least one is true:<br>● ICD-9: 435<br>● ICD-10: G45<br>● ICPC-2: K89
Type 1 diabetes mellitus	At least one is true:<br>● ICD-9: 2500[13], 2501[13], 2502[13], 2503[13], 2504[13], 2505[13], 2506[13], 2507[13], 2508[13], 2509[13]<br>● ICD-10: E10<br>● ICPC-2: T89
Type 2 diabetes mellitus	At least one is true:<br>● HbA1c level ≥ 6.5%<br>● Plasma glucose ≥ 200 mg/mL<br>● Glucose lowering drugs except metformin in isolation<br>● Excluding Type 1 Diabetes Mellitus
Unstable angina	At least one is true:<br>● ICD-9: 411<br>● ICD-10: I200<br>● ICPC-2: K74

UA - Unstable angina; MI - Myocardial infarction; SA - Stable angina; STK - Stroke; PAD - Peripheral arterial disease; LVEF - Left ventricular ejection fraction. **Diagnosis:** Primary care diagnoses are coded using ICPC-2 codes. Hospital diagnosis are coded using ICD-9 codes for all visits, except for hospitalization visits since 01-01-2017 that are coded using ICD-10; **Measurement:** Data comes from measurements performed at any venue care site; **Medication:** All prescribed medications from identified persons that match ATC codes. May include medications prescribed by providers outside ULSM.

Table S6

Rate of LDL-C Control Events per 100 Patient-Years by Lipid-Lowering Therapy (LLT) During Follow-Up.

Follow-up	Lipid-Lowering Therapy	Number episodes	Events (Ev)	Patient-Year (PY)	Ev/100PY
**150 days**	Low Intensity	26 751	132	10 986	1.20
	Moderate Intensity	296 786	2 072	121 865	1.70
	High Intensity	37 659	356	15 460	2.30
	Low Intensity + Ezetimibe	351	3	144	2.08
	Moderate Intensity + Ezetimibe	4 896	53	2 010	2.64
	High Intensity Statin + Ezetimibe	3 312	45	1 359	3.31
	**Total**	**369 755**	**2 661**	**151 824**	**1.75**
**180 days**	Low Intensity	26 751	272	13 161	2.07
	Moderate Intensity	296 786	4 344	145 940	2.98
	High Intensity	37 659	748	18 501	4.04
	Low Intensity + Ezetimibe	351	6	172	3.49
	Moderate Intensity + Ezetimibe	4 896	107	2 405	4.45
	High Intensity Statin + Ezetimibe	3 312	108	1 624	6.65
	**Total**	**369 755**	**5 585**	**181 803**	**3.07**
**210 days**	Low Intensity	26 751	372	15 321	2.43
	Moderate Intensity	296 786	5 914	169 802	3.48
	High Intensity	37 659	1 011	21 506	4.70
	Low Intensity + Ezetimibe	351	7	201	3.48
	Moderate Intensity + Ezetimibe	4 896	155	2 795	5.55
	High Intensity Statin + Ezetimibe	3 312	143	1 885	7.59
	**Total**	**369 755**	**7 602**	**211 510**	**3.59**
**240 days**	Low Intensity	26 751	436	17 470	2.50
	Moderate Intensity	296 786	6 957	193 521	3.59
	High Intensity	37 659	1 237	24 487	5.05
	Low Intensity + Ezetimibe	351	9	229	3.93

Follow-up	Lipid-Lowering Therapy	Number episodes	Events (Ev)	Patient-Year (PY)	Ev/100PY
	Moderate Intensity + Ezetimibe	4 896	186	3 182	5.85
	High Intensity Statin + Ezetimibe	3 312	166	2 142	7.75
	**Total**	**369 755**	**8 991**	**241 031**	**3.73**
**270 days**	Low Intensity	26 751	484	19 610	2.47
	Moderate Intensity	296 786	7 894	217 115	3.64
	High Intensity	37 659	1 433	27 442	5.22
	Low Intensity + Ezetimibe	351	10	257	3.89
	Moderate Intensity + Ezetimibe	4 896	208	3 565	5.83
	High Intensity Statin + Ezetimibe	3 312	189	2 397	7.88
	**Total**	**369 755**	**10 218**	**270 386**	**3.78**
**300 days**	Low Intensity	26 751	546	21 740	2.51
	Moderate Intensity	296 786	8 700	240 594	3.62
	High Intensity	37 659	1 592	30 373	5.24
	Low Intensity + Ezetimibe	351	11	284	3.87
	Moderate Intensity + Ezetimibe	4 896	224	3 947	5.68
	High Intensity Statin + Ezetimibe	3 312	206	2 649	7.78
	**Total**	**369 755**	**11 279**	**299 587**	**3.76**
**330 days**	Low Intensity	26 751	619	23 860	2.59
	Moderate Intensity	296 786	9 672	263 955	3.66
	High Intensity	37 659	1 780	33 281	5.35
	Low Intensity + Ezetimibe	351	11	312	3.53
	Moderate Intensity + Ezetimibe	4 896	247	4 325	5.71
	High Intensity Statin + Ezetimibe	3 312	221	2 899	7.62
	**Total**	**369 755**	**12 550**	**328 632**	**3.82**
**360 days**	Low Intensity	26 751	685	25 968	2.64
	Moderate Intensity	296 786	10 845	287 171	3.78
	High Intensity	37 659	1 991	36 159	5.51
	Low Intensity + Ezetimibe	351	11	339	3.24
	Moderate Intensity + Ezetimibe	4 896	271	4 700	5.77

Follow-up	Lipid-Lowering Therapy	Number episodes	Events (Ev)	Patient-Year (PY)	Ev/100PY
	High Intensity Statin + Ezetimibe	3 312	244	3 145	7.76
	**Total**	**369 755**	**14 047**	**357 482**	**3.93**

Table S7

Model summary for 150 days follow-up

	HR	CI Low	CI High
**Age**			
40 - 69 years	Reference		
70 - 85 years	1.32	1.19	1.45
**Sex**			
Female	Reference		
Male	2.56	2.30	2.85
**LLT prescription pattern**			
Moderate Intensity	Reference		
High Intensity + Ezetimibe	1.82	1.34	2.47
Low Intensity + Ezetimibe	1.20	0.28	5.18
Moderate Intensity + Ezetimibe	1.48	1.11	1.98
Low Intensity	0.69	0.55	0.85
High Intensity	1.31	1.15	1.49
**Comorbidities**			
Obesity	1.03	0.93	1.14
Hypertension	0.93	0.79	1.09
Alcohol or drug abuse	0.84	0.74	0.96
Type 1 diabetes mellitus	1.20	1.01	1.42
Type 2 diabetes mellitus	0.88	0.80	0.97
Atrial fibrillation	1.09	0.93	1.27
Primary hypertriglyceridemia	1.21	1.07	1.38
Stroke	1.16	1.02	1.32
Myocardial infarction	1.05	0.90	1.22
Peripheral artery disease	0.91	0.78	1.06

	HR	CI Low	CI High
Unstable angina	0.86	0.72	1.01
Heart failure	1.37	1.18	1.59
Mental health disorder	0.84	0.76	0.92
Estimated glomerular filtration rate < 60ml/min	1.28	1.14	1.45

CI - confidence interval; HR - hazard ratio; LLT - lipid lowering therapy

Table S8

Model summary for 180 days follow-up

	HR	CI Low	CI High
**Age**			
40 - 69 years	Reference		
70 – 85 years	1.32	1.22	1.43
**Sex**			
Female	Reference		
Male	2.62	2.41	2.86
**LLT prescription pattern**			
Moderate Intensity	Reference		
High Intensity + Ezetimibe	2.16	1.73	2.71
Low Intensity + Ezetimibe	1.15	0.45	2.91
Moderate Intensity + Ezetimibe	1.45	1.17	1.81
Low Intensity	0.67	0.57	0.80
High Intensity	1.33	1.21	1.47
**Comorbidities**			
Obesity	1.04	0.96	1.12
Hypertension	0.98	0.87	1.11
Alcohol or drug abuse	0.84	0.76	0.93
Type 1 diabetes mellitus	1.25	1.10	1.42
Type 2 diabetes mellitus	0.85	0.79	0.92
Atrial fibrillation	1.07	0.95	1.22
Primary hypertriglyceridemia	1.15	1.04	1.27
Stroke	1.10	0.98	1.22
Myocardial infarction	1.02	0.91	1.15
Peripheral artery disease	0.86	0.76	0.97
Unstable angina	0.86	0.75	0.99

	HR	CI Low	CI High
Heart failure	1.25	1.11	1.41
Mental health disorder	0.85	0.79	0.92
Estimated glomerular filtration rate < 60ml/min	1.24	1.13	1.37

CI, confidence interval; HR, hazard ratio; LLT, lipid lowering therapy

Table S9

Model summary for 210 days follow-up

	HR	CI Low	CI High
**Age**			
40 - 69 years	Reference		
70 – 85 years	1.32	1.22	1.41
**Sex**			
Female	Reference		
Male	2.62	2.43	2.83
**LLT prescription pattern**			
Moderate Intensity	Reference		
High Intensity + Ezetimibe	2.07	1.68	2.54
Low Intensity + Ezetimibe	0.97	0.42	2.27
Moderate Intensity + Ezetimibe	1.54	1.27	1.86
Low Intensity	0.68	0.58	0.79
High Intensity	1.31	1.20	1.43
**Comorbidities**			
Obesity	1.04	0.97	1.12
Hypertension	0.99	0.88	1.11
Alcohol or drug abuse	0.87	0.79	0.95
Type 1 diabetes mellitus	1.21	1.08	1.37
Type 2 diabetes mellitus	0.83	0.77	0.89
Atrial fibrillation	1.12	1.00	1.25
Primary hypertriglyceridemia	1.18	1.08	1.28

	HR	CI Low	CI High
Stroke	1.12	1.02	1.24
Myocardial infarction	1.03	0.92	1.15
Peripheral artery disease	0.86	0.77	0.96
Unstable angina	0.89	0.78	1.01
Heart failure	1.26	1.13	1.40
Mental health disorder	0.88	0.82	0.94
Estimated glomerular filtration rate < 60ml/min	1.30	1.20	1.41

CI, confidence interval; HR, hazard ratio; LLT, lipid lowering therapy

Table S10

Model summary for 240 days follow-up

	HR	CI Low	CI High
**Age**			
40 - 69 years	Reference		
70 – 85 years	1.30	1.22	1.39
**Sex**			
Female	Reference		
Male	2.60	2.41	2.79
**LLT prescription pattern**			
Moderate Intensity	Reference		
High Intensity + Ezetimibe	2.01	1.65	2.46
Low Intensity + Ezetimibe	1.07	0.49	2.33
Moderate Intensity + Ezetimibe	1.56	1.30	1.86
Low Intensity	0.67	0.58	0.78
High Intensity	1.35	1.24	1.47
**Comorbidities**			
Obesity	1.02	0.95	1.09
Hypertension	1.00	0.90	1.11
Alcohol or drug abuse	0.87	0.79	0.95
Type 1 diabetes mellitus	1.30	1.16	1.45
Type 2 diabetes mellitus	0.86	0.81	0.92
Atrial fibrillation	1.12	1.01	1.25
Primary hypertriglyceridemia	1.20	1.10	1.30
Stroke	1.14	1.04	1.25
Myocardial infarction	1.03	0.93	1.14
Peripheral artery disease	0.87	0.78	0.96

	HR	CI Low	CI High
Unstable angina	0.91	0.81	1.03
Heart failure	1.32	1.20	1.46
Mental health disorder	0.90	0.84	0.96
Estimated glomerular filtration rate < 60ml/min	1.29	1.20	1.40

CI, confidence interval; HR, hazard ratio; LLT, lipid lowering therapy

Table S11

Model summary for 270 days follow-up

	HR	CI Low	CI High
**Age**			
40 - 69 years	Reference		
70 – 85 years	1.28	1.20	1.37
**Sex**			
Female	Reference		
Male	2.55	2.37	2.74
**LLT prescription pattern**			
Moderate Intensity	Reference		
High Intensity + Ezetimibe	1.97	1.62	2.40
Low Intensity + Ezetimibe	1.05	0.48	2.27
Moderate Intensity + Ezetimibe	1.52	1.28	1.80
Low Intensity	0.66	0.58	0.76
High Intensity	1.36	1.25	1.47
**Comorbidities**			
Obesity	1.01	0.95	1.08
Hypertension	1.01	0.91	1.12
Alcohol or drug abuse	0.89	0.81	0.96
Type 1 diabetes mellitus	1.31	1.18	1.46
Type 2 diabetes mellitus	0.88	0.83	0.94
Atrial fibrillation	1.15	1.04	1.27
Primary hypertriglyceridemia	1.21	1.12	1.31
Stroke	1.15	1.05	1.25
Myocardial infarction	1.08	0.98	1.19
Peripheral artery disease	0.88	0.79	0.97

	HR	CI Low	CI High
Unstable angina	0.92	0.82	1.04
Heart failure	1.34	1.22	1.48
Mental health disorder	0.92	0.86	0.98
Estimated glomerular filtration rate < 60ml/min	1.33	1.23	1.43

CI, confidence interval; HR, hazard ratio; LLT, lipid lowering therapy

Table S12

Model summary for 300 days follow-up

	HR	CI Low	CI High
**Age**			
40 - 69 years	Reference		
70 – 85 years	1.28	1.20	1.37
**Sex**			
Female	Reference		
Male	2.50	2.33	2.68
**LLT prescription pattern**			
Moderate Intensity	Reference		
High Intensity + Ezetimibe	1.93	1.59	2.35
Low Intensity + Ezetimibe	1.04	0.51	2.13
Moderate Intensity + Ezetimibe	1.48	1.25	1.75
Low Intensity	0.68	0.59	0.77
High Intensity	1.36	1.26	1.47
**Comorbidities**			
Obesity	1.01	0.95	1.08
Hypertension	1.00	0.90	1.10
Alcohol or drug abuse	0.89	0.82	0.97
Type 1 diabetes mellitus	1.35	1.22	1.50
Type 2 diabetes mellitus	0.91	0.85	0.97
Atrial fibrillation	1.16	1.05	1.28
Primary hypertriglyceridemia	1.22	1.13	1.32
Stroke	1.15	1.06	1.26
Myocardial infarction	1.09	0.98	1.20
Peripheral artery disease	0.90	0.81	0.99

	HR	CI Low	CI High
Unstable angina	0.93	0.83	1.04
Heart failure	1.37	1.25	1.51
Mental health disorder	0.93	0.87	0.99
Estimated glomerular filtration rate < 60ml/min	1.35	1.25	1.45

CI, confidence interval; HR, hazard ratio; LLT, lipid lowering therapy

Table S13

Model summary for 330 days follow-up

	HR	CI Low	CI High
**Age**			
40 - 69 years	Reference		
70 – 85 years	1.27	1.19	1.35
**Sex**			
Female	Reference		
Male	2.44	2.28	2.61
**LLT prescription pattern**			
Moderate Intensity	Reference		
High Intensity + Ezetimibe	1.86	1.53	2.25
Low Intensity + Ezetimibe	0.94	0.46	1.92
Moderate Intensity + Ezetimibe	1.46	1.24	1.73
Low Intensity	0.69	0.61	0.79
High Intensity	1.36	1.26	1.48
**Comorbidities**			
Obesity	1.02	0.96	1.08
Hypertension	0.99	0.90	1.10
Alcohol or drug abuse	0.89	0.82	0.96
Type 1 diabetes mellitus	1.37	1.24	1.52
Type 2 diabetes mellitus	0.93	0.87	0.99
Atrial fibrillation	1.19	1.08	1.31
Primary hypertriglyceridemia	1.22	1.13	1.32
Stroke	1.16	1.07	1.26
Myocardial infarction	1.10	1.00	1.22
Peripheral artery disease	0.92	0.84	1.02

	HR	CI Low	CI High
Unstable angina	0.92	0.82	1.03
Heart failure	1.37	1.25	1.50
Mental health disorder	0.93	0.88	0.99
Estimated glomerular filtration rate < 60ml/min	1.36	1.27	1.47

CI, confidence interval; HR, hazard ratio; LLT, lipid lowering therapy

Table S14

Model summary for 360 days follow-up

	HR	CI Low	CI High
**Age**			
40 - 69 years	Reference		
70 – 85 years	1.27	1.19	1.35
**Sex**			
Female	Reference		
Male	2.44	2.28	2.61
**LLT prescription pattern**			
Moderate Intensity	Reference		
High Intensity + Ezetimibe	1.84	1.52	2.23
Low Intensity + Ezetimibe	0.84	0.41	1.72
Moderate Intensity + Ezetimibe	1.44	1.22	1.69
Low Intensity	0.68	0.60	0.77
High Intensity	1.36	1.26	1.47
**Comorbidities**			
Obesity	1.01	0.95	1.08
Hypertension	0.99	0.90	1.09
Alcohol or drug abuse	0.88	0.81	0.96
Type 1 diabetes mellitus	1.40	1.27	1.55
Type 2 diabetes mellitus	0.94	0.88	1.00
Atrial fibrillation	1.18	1.07	1.30
Primary hypertriglyceridemia	1.23	1.14	1.33
Stroke	1.16	1.07	1.26
Myocardial infarction	1.10	0.99	1.21
Peripheral artery disease	0.92	0.84	1.01

	HR	CI Low	CI High
Unstable angina	0.92	0.82	1.03
Heart failure	1.36	1.24	1.49
Mental health disorder	0.94	0.88	1.00
Estimated glomerular filtration rate < 60ml/min	1.35	1.26	1.45

CI, confidence interval; HR, hazard ratio; LLT, lipid lowering therapy
